# CD47 antisense oligonucleotide treatment attenuates obesity and its-associated metabolic dysfunction

**DOI:** 10.1038/s41598-023-30006-2

**Published:** 2023-02-16

**Authors:** Taesik Gwag, Dong Li, Eric Ma, Zhenheng Guo, Ying Liang, Shuxia Wang

**Affiliations:** 1 Department of Pharmacology and Nutritional Sciences, University of Kentucky, Wethington Bldg. Room 583, 900 S. Limestone Street, Lexington, KY 40536 USA; 2 Department of Toxicology and Cancer Biology, University of Kentucky, Lexington, KY 40536 USA; 3 Lexington Veterans Affairs Medical Center, Lexington, KY 40502 USA

**Keywords:** Drug discovery, Physiology, Diseases

## Abstract

Previous study from our lab has revealed a new role of CD47 in regulating adipose tissue function, energy homeostasis and the development of obesity and metabolic disease in CD47 deficient mice. In this study, the therapeutic potential of an antisense oligonucleotide (ASO) targeting to CD47 in obesity and its-associated complications was determined in two obese mouse models (diet induced and genetic models). In diet induced obesity, male C57BL6 mice were fed with high fat (HF) diet to induce obesity and then treated with CD47ASO or control ASO for 8 weeks. In genetic obese mouse model*,* male six-week old *ob/ob* mice were treated with ASOs for 9 weeks. We found that CD47ASO treatment reduced HF diet-induced weight gain, decreased fat mass, prevented dyslipidemia, and improved glucose tolerance. These changes were accompanied by reduced inflammation in white adipose tissue and decreased hepatic steatosis. This protection was also seen in CD47ASO treated *ob/ob* mice. Mechanistically, CD47ASO treatment increased mice physical activity and energy expenditure, contributing to weight loss and improved metabolic outcomes in obese mice. Collectively, these findings suggest that CD47ASO might serve as a new treatment option for obesity and its-associated metabolic complications.

## Introduction

CD47 is an integral glycoprotein cell receptor with a molecular weight of 45–55 kDa^1,2^. It is widely expressed at lower levels by most healthy cells throughout the body^3,4^. Many well-established functions (e.g. immunity, self-recognition, cellular adhesion, and vascular tone) are dependent on the ligand or partner receptor associated with CD47^2,5–10^. CD47 has been shown to couple to heterotrimeric G_i_α and inhibits adenylyl cyclase and then cAMP signaling^8,9,11^. CD47 also inhibits soluble guanylyl cyclase activity to suppress cGMP signaling^2,10^. Both of cAMP and cGMP signaling pathways regulate adipocyte function and impact metabolic health. Accordingly, we have revealed a novel role for CD47 in regulating adipocyte function and its contribution to diet or aging-related obesity and metabolic disorders^12–14^.

Our previous studies showed that genetic deletion (global) of CD47 protected young adult mice from high fat diet-induced obesity by displaying decreased weight gain and reduced adiposity, reduced adipose tissue inflammation, and improved glucose tolerance and insulin sensitivity. This protection is associated with increased brown fat activation, increased lipid utilization and energy expenditure^12,13^. In addition to protecting mice from diet-induced obesity, genetic deletion of CD47 also protects mice from natural aging-associated obesity and glucose intolerance^14–16^. Under normal chow diet conditions, old male CD47 deficient mice had reduced visceral fat mass and improved glucose tolerance. These mice also had increased energy expenditure and better cold tolerance, accompanied by increased white adipose tissue browning and brown adipose tissue (BAT) activity. Collectively, these studies support that CD47 might be a potential target for metabolic disorders of obesity. Thus, in this study, the therapeutic effect of CD47 antisense oligonucleotides (ASO) on obesity and its associated metabolic dysfunction was determined in two obese mouse models (high fat diet induced obesity or genetic obesity models).

Interestingly, in this study, CD47ASO treatment did not stimulate white fat browning or brown fat activity in obese mice at the current dosage. Instead, CD47ASO treatment increased mice physical activity and energy expenditure. It also reduced inflammation in white adipose tissue. All of these effects contribute to the weight loss and improved metabolic outcomes as seen from CD47ASO treated mice.

## Results

### CD47ASO treatment reduced body weight, adiposity, plasma lipid levels and improved glucose tolerance and fatty liver disease in mice with established obesity

**Figure 1:**
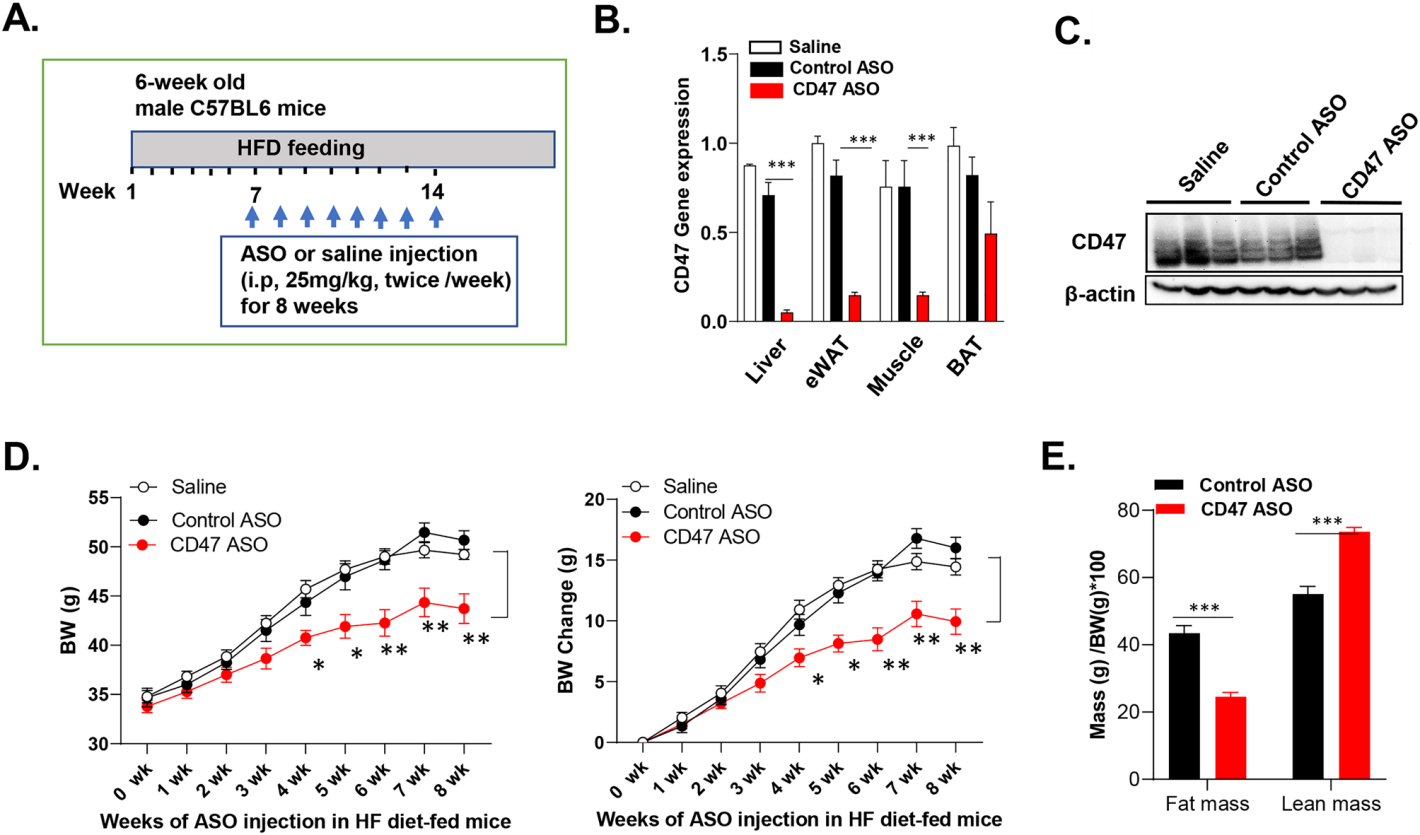
CD47ASO treatment induced weight loss and reduced adiposity in diet-induced obese mice. (**A**) Schematic diagram of experimental setting; (**B**) CD47 mRNA levels from different tissues after saline or ASO treatment by qPCR; (**C**) CD47 protein levels in liver by immunoblotting (cropped blots are displayed); (**D**) Weekly body weight and body weight change after ASO treatment; and (**E**) Body composition (fat mass and lean mass) detected by EchoMRI. HFD: high fat diet. Data are represented as mean ± SE (n = 4–7 mice/group). *P < 0.05, **P < 0.01, ***P < 0.001.

**Figure 2:**
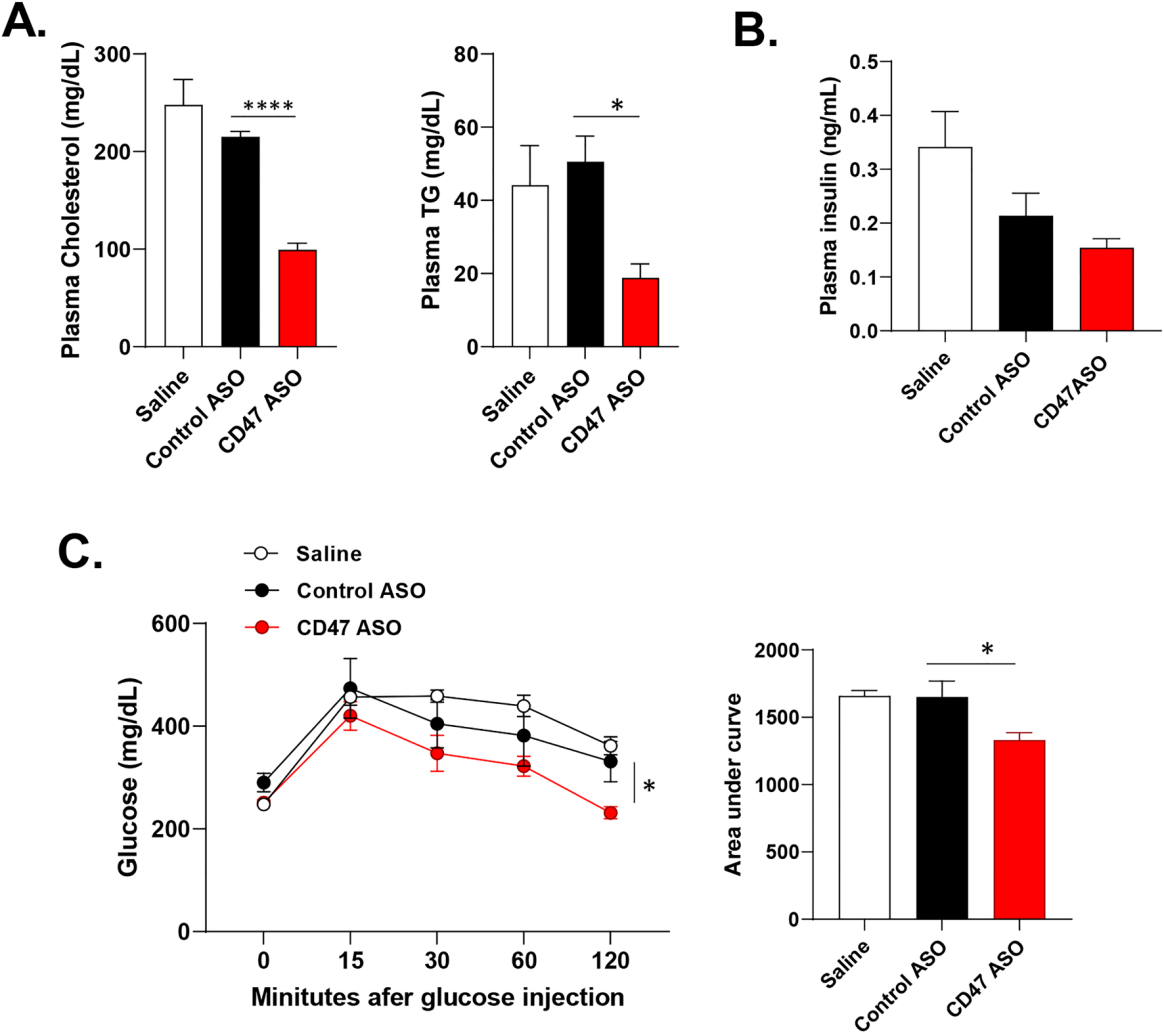
CD47ASO treatment reduced hyperlipidemia and improved glucose tolerance in diet-induced obese mice. (**A**) Plasma total cholesterol and triglyceride levels; (**B**) plasma insulin levels; (**C**) Glucose tolerance test (GTT) and area under the curve were analyzed. Data are represented as mean ± SE (n = 4–7 mice/group). *P < 0.05, ****P < 0.0001.

**Figure 3:**
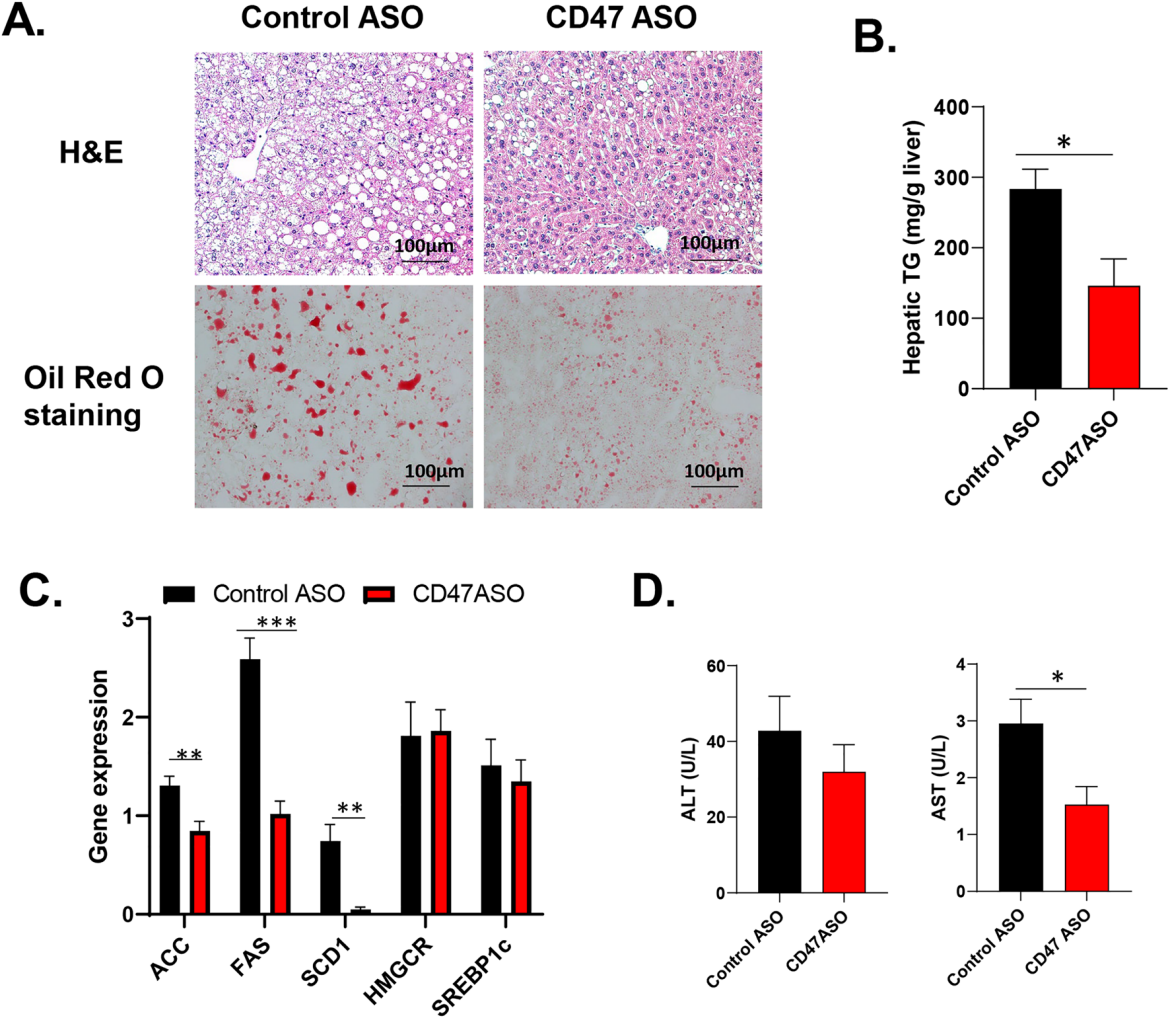
CD47ASO treatment reduced hepatic steatosis in diet-induced obese mice. (**A**) Representative H&E staining and Oil red O staining of liver sections from control ASO or CD47 ASO treated mice; (**B**) Liver triglyceride levels; (**C**) Liver gene expression by qPCR; (**D**) Plasma ALT and AST levels. Data are represented as mean ± SE (n = 4–7 mice/group). *P < 0.05, **P < 0.01, ***P < 0.001.

To test the therapeutic effect of CD47ASO on obesity and its-associated metabolic dysfunction, we utilized two obese mouse model in this study. One is the high fat diet (60% fat) induced obesity mouse model (DIO model), a well-established obese mouse model in the field. After 6 weeks of HF diet feeding, male C57BL6 mice developed obesity and glucose intolerance (Fig. S1). CD47ASO or control ASO were then administered into these obese mice for 8 weeks with continuous HF feeding (Fig. 1A). Saline group was also included to examine the non-specific effect that might be induced by control ASO. As shown in Fig. 1B,C, CD47 ASO efficiently knockdown CD47 in metabolic tissues such as liver, skeletal muscle or fat tissues. CD47ASO treatment significantly reduced HF diet induced weight gain starting after 4 weeks of CD47 ASO treatment. Control ASO treatment did not induce weight loss as compared to saline group (Fig. 1D). Fat mass was also significantly reduced, but lean mass was increased in CD47ASO treated mice group (Fig. 1E). The weight loss effect was also confirmed by using two additional CD47ASOs (targeting to different sequence of mouse CD47) (Fig. S2). Plasma lipid levels including triglyceride and cholesterol were reduced by CD47ASO treatment (Fig. 2A). Plasma insulin levels were comparable among all groups (Fig. 2B). However, glucose tolerance was improved in CD47ASO treated mice (Fig. 2C). In addition, CD47ASO treatment reduced hepatic steatosis as demonstrated by reduced liver Oil Red O staining and liver triglyceride measurement (Fig. 3A,B), which was associated with reduced expression of lipid synthesis genes including FAS, ACC and SCD1 (Fig. 3C). Accordingly, plasma ALT and AST levels were reduced in CD47ASO treated mice (Fig. 3D).

In addition to DIO mouse model, genetic obese mouse model (*ob/ob*) mice were also used in the study. Six-week old male *ob/ob* mice received saline, control ASO, and CD47ASO treatment for 9 weeks. We found that *ob/ob* mice showed significant weight loss after 5 weeks of CD47ASO treatment; while control ASO treatment did not induce mice weight loss. CD47ASO treated *ob/ob* mice displayed reduced adiposity, accompanied with improved glucose tolerance and fatty liver disease as compared to control ASO group (Fig. S3). Together, by utilization of two established obesity mouse models, our data demonstrated that CD47ASO treatment induced obese mice to undergo weight loss and improved blood glucose and lipid homeostasis.

### CD47 ASO treatment reduced inflammation in white adipose tissue in mice with established obesity.

**Figure 4:**
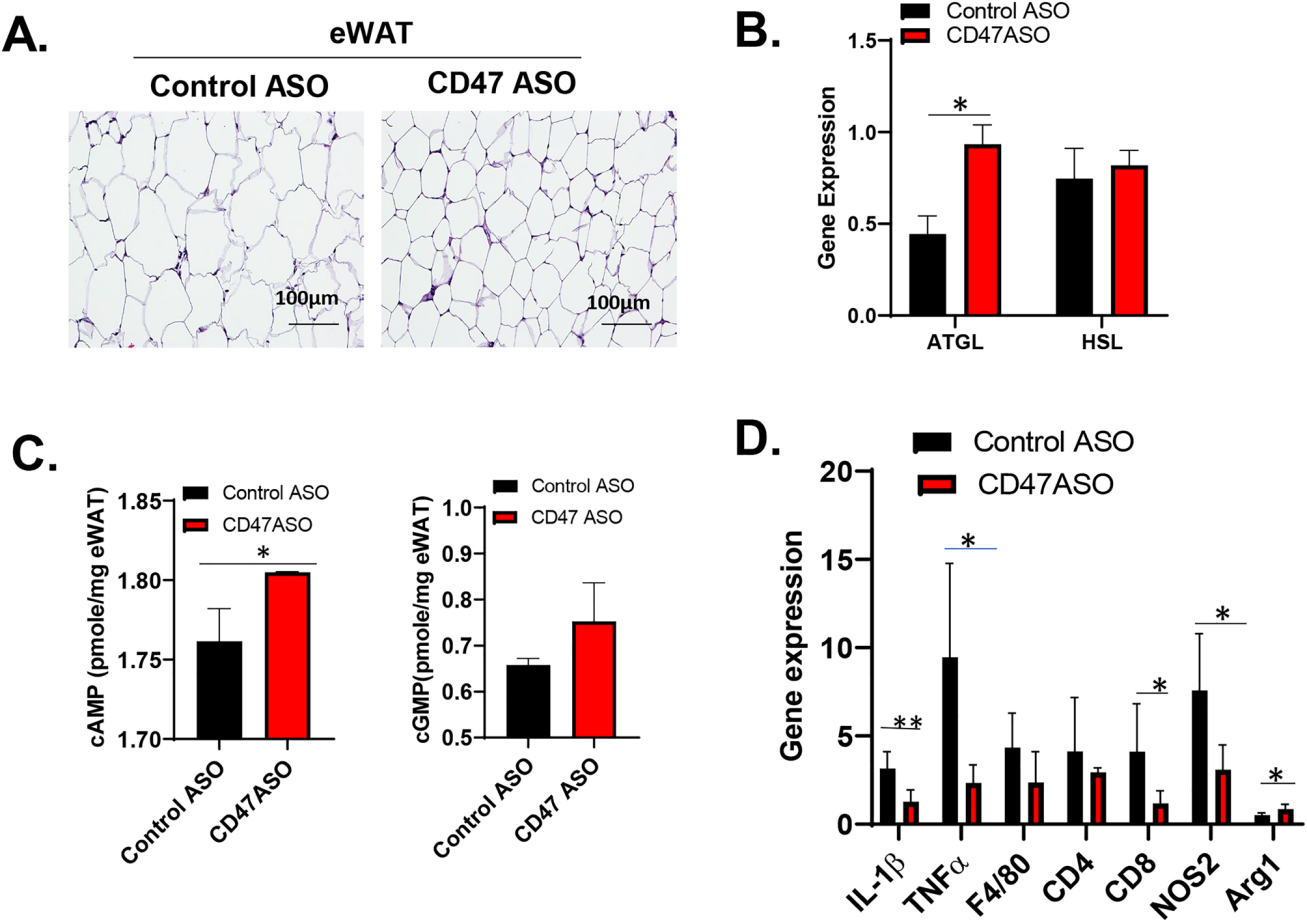
CD47ASO treatment reduced white adipose tissue inflammation in diet-induced obese mice (**A**). Representative H&E staining of eWAT (epidydimal white adipose tissue) from control ASO and CD47ASO treated mice. (**B**) Expression of lipolysis related genes (ATGL and HSL) by qPCR and normalized to β-actin levels; (**C**) cAMP and cGMP levels; (**D**) Expression of inflammation related genes by qPCR and normalized to β-actin levels. Data are represented as mean ± SE (n = 4–7 mice/group). *P < 0.05, **P < 0.01.

To determine the involved mechanisms of CD47ASO mediated weight loss and improved glucose tolerance, we analyzed epidydimal white fat tissue (eWAT). As shown in Fig. 4A, eWAT from CD47ASO treated mice had smaller size of adipocytes. Further qPCR analysis of eWAT showed that CD47ASO treatment increased expression of lipolysis genes (e.g. ATGL) (Fig. 4B), which was accompanied by increased cAMP levels in EAT (Fig. 4C). This suggests that increased lipolysis of eWAT may contribute to the reduced adiposity in CD47ASO treated mice. CD47ASO treatment also reduced the expression of mRNA for pro-inflammatory cytokines (e.g. TNF-α and IL-1β) in eWAT (Fig. 4D). Interestingly, F4/80 expression (macrophage marker) was comparable between control ASO and CD47ASO group. Arg1 expression (M2 macrophage marker) was increased; while NOS2 (M1 macrophage marker) was reduced by CD47ASO treatment. This suggests that CD47ASO treatment may induce adipose tissue macrophage phenotype switch from proinflammatory M1 to anti-inflammatory M2. In addition, CD8 expression (a marker for effector T cell) was reduced by CD47ASO treatment. Together, these data suggest that CD47 ASO treatment reduced inflammation in white adipose tissue, partially through regulation of adipose tissue macrophage phenotype and/or T cell subpopulations^17–19^.

### CD47 ASO treated mice had increased energy expenditure

**Figure 5:**
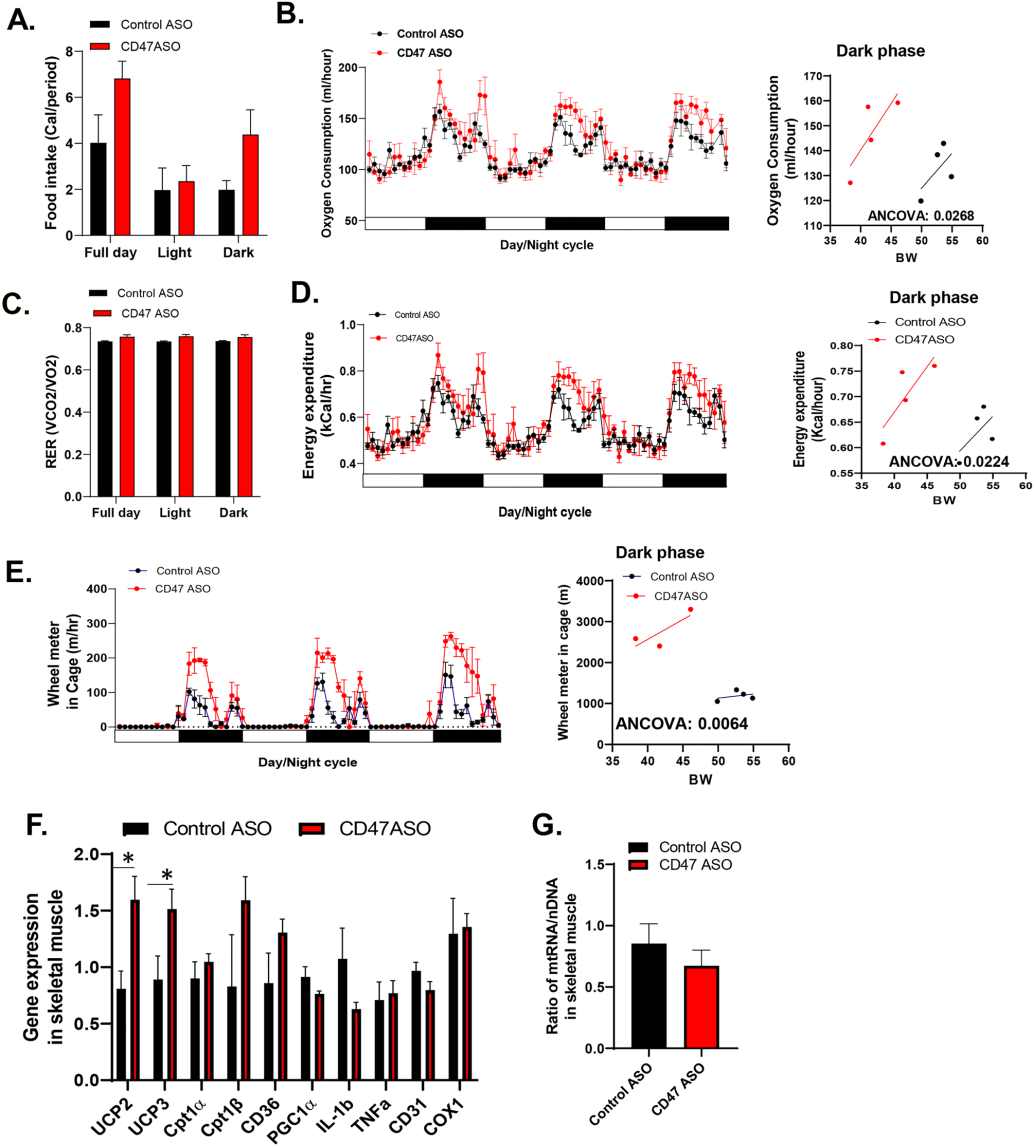
CD47 ASO treatment increased activity in diet-induced obese mice. Mice were housed in Sable Promethion system for indirect calorimetry analysis. (**A**) Food intake; (**B**) Oxygen consumption of three day/night cycles and regression plot of dark period oxygen consumption versus body weight (BW); (**C**) RER (VCO2/VO2); (**D**) Energy expenditure of three day/night cycles and regression plot of dark period energy expenditure versus BW; (**E**) Voluntary wheel running distance of three day/night cycles and regression plot of dark period wheel running distance versus BW; (**F**) Skeletal muscle gene expression by qPCR; (**G**) Skeletal muscle DNA copy number by qPCR. Data are presented as mean ± SE (n = 4 mice /group). *P < 0.05.

To determine how CD47ASO treatment affected energy balance, mouse indirect calorimetry was performed by utilization of Sable Promethion System including in-cage wheels. We found that CD47ASO treated mice had a trend of increased food intake at dark phase (Fig. 5A). RER (VCO_2_/VO_2_) was comparable between control ASO and CD47 ASO treatment group (Fig. 5C). However, oxygen consumption and energy expenditure were significantly increased by CD47ASO treatment, especially at dark phase demonstrated by analysis of covariance (ANCOVA) between oxygen consumption/energy expenditure and body weight (Fig. 5B and D). Interestingly, CD47ASO treated mice also displayed significantly increased voluntary wheel running distances (Fig. 5E), suggesting increased physical activity. This was accompanied by increased expression of uncoupling protein 2 and 3 (UCP2 and UCP3) in skeletal muscle (Fig. 5F). UCP3 is a member of the mitochondrial anion carrier protein family mainly expressed in skeletal muscle and plays a role in fatty acid oxidation and energy balance^20^. Other fatty acid oxidation or fatty acid uptake related genes such as Cpt1β (carnitine palmitoyltransferase 1, located in the outer of mitochondria membrane and an indispensable enzyme of fatty acid oxidation) or CD36 had a trend of increase in skeletal muscle from CD47ASO treated mice. These data support a role of CD47ASO treatment in regulating skeletal muscle functions. This effect might be independent of mitochondria number in skeletal muscle since PCR data showed that mitochondria DNA copy number in skeletal muscle was comparable between control ASO and CD47 ASO groups (Fig. 5G).

In addition to skeletal muscle, beige and brown fat-mediated thermogenesis could also contribute to increased energy expenditure as seen from CD47ASO treated mice. Therefore, the phenotype of inguinal white fat (iWAT) and brown fat tissue (BAT) from CD47ASO treated DIO mice was also examined. As shown in Fig. S4, iWAT from CD47ASO treated mice had smaller size of adipocytes. However, expression of genes related to white fat browning (UCP1), inflammation (IL-1β and TNF-α) or lipolysis (ATGL and HSL) were comparable between control and CD47ASO groups. As for BAT, expression of genes related to brown fat function such as UCP1, PGC1α, CPT1α, et al. were not significantly altered by CD47ASO treatment. Moreover, CD47ASO treated mice showed similar cold intolerance as control mice. Collectively, these data suggest that at the current CD47ASO dosage, with moderately downregulated brown fat CD47 levels, brown fat activity was not significantly altered. So, BAT may not contribute to the increased energy expenditure seen from CD47ASO treated mice; while increased physical activity is the main contributor.

## Discussion

In this study, the therapeutic potential of antisense oligonucleotide (ASO) targeting to CD47 in obesity and metabolic disease was determined. We demonstrate that CD47ASO treatment induces weight loss, decreases fat mass, and improves glucose homeostasis and fatty liver disease in two obese mouse models (DIO and *ob/ob* mice). These metabolic beneficial effects are associated with increased energy expenditure mainly from skeletal muscle and reduced inflammation in white adipose tissue. Our data suggest that CD47ASO might be a new therapeutic option for obesity and its associated metabolic dysfunction.

One finding of this study is that CD47ASO treatment induced obese mice weight loss, which was associated with increased energy utilization. Previously, we showed that *CD47* deficiency protected mice from high fat diet induced obesity, which was attributed to the increased energy expenditure driven by brown adipose tissue (BAT) activity^13^. BAT expends energy through UCP1-mediated uncoupling, resulting in heat production or thermogenesis^21,22^. Therefore, first, we analyzed BAT function in ASOs treated mice. Interestingly, at the current dosage of CD47ASO (i.p. at 25 mg/kg, twice a week), BAT activity was not significantly affected. We found that the expression of UCP1, CPT1 (a carnitine palmitoyltransferase 1, the first and rate-limiting step for fatty acid transporting into the mitochondria for utilization^23^), or PGC1α in BAT were not altered by CD47ASO treatment. CD47ASO treated mice also showed similar cold intolerance as control mice. In addition, there was no white fat browning revealed in CD47ASO treated mice. These data suggest that at the current CD47ASO dosage, brown fat-mediated thermogenesis was not altered by CD47ASO treatment and thus BAT may not contribute to the increased energy expenditure as shown in CD47ASO mice. A higher dosage of CD47ASO may need to be tested in the future study.

Next, we determined the contribution of skeletal muscle to increased energy expenditure in CD47ASO treated mice. A previous study showed that CD47 deficient mice had enhanced treadmill endurance and their skeletal muscle had greater number of mitochondria as compared to wild type mice^16^. CD47 also plays a role in regulating aged muscle stem cell regeneration capacity^24^. In the current study, we found that CD47ASO treated mice had increased physical activity demonstrated by increased voluntary wheel running distance in metabolic cages. Further analysis of skeletal muscle gene expression profile, we found that CD47ASO treatment did not increase genes related to mitochondria biogenesis (e.g. PGC1 alpha) in skeletal muscle. Consistently, mitochondria DNA copy number in skeletal muscle was comparable between control ASO and CD47 ASO groups. However, the expression of uncoupling proteins UCP2 and UCP3 in skeletal muscle was significantly upregulated in CD47ASO treated mice. UCP2 and UCP3 have high sequence homology to UCP1^25,26^. Unlike UCP1 in BAT, studies suggest that UCP2 and UCP3 do not mediate thermogenesis^27^. UCP2 is expressed in many tissues including skeletal muscle; while UCP3 is predominantly expressed in skeletal muscle in humans and rodents^25,28^ and plays a role in fatty acid oxidation and body metabolism^27,29,30^. UCP3 also involves in the effect of physical activity on energy expenditure^20^. Increasing UCP3 expression in skeletal muscle stimulates proton leak across the inner mitochondrial membrane and increases oxygen consumption in isolated mitochondria^31^. Also, in vivo data showed that UCP3 in skeletal muscle is able to uncouple respiration from ATP production^32,33^. This process results in lower energy efficiency and possibly increased energy expenditure. Consistently, transgenic mice with overexpression of UCP3 in skeletal muscle were lean^31^ and insulin sensitive^34^. Based on these observations, we speculate that increased expression of UCP3 in skeletal muscle may mediate CD47ASO treatment induced increase in energy expenditure. This warrants further investigation in the future.

CD47 has well-known immune-regulatory functions^35–39^. In this study, the effect of CD47ASO treatment on white adipose tissue inflammation in obese mice was examined. It is well-known that obesity is associated with low-grade chronic inflammation. Visceral adipose tissue has been suggested to be the primary source of cytokine and adipokine release within obesity-associated inflammation. It is marked by the accumulation of macrophages, T cells and other immune cells in adipose tissue, leading to the development of glucose tolerance and insulin resistance^37,40,41^. We found that CD47ASO treatment significantly reduced pro-inflammatory cytokine production in eWAT, which was accompanied by improved whole body glucose tolerance. This CD47ASO-mediated anti-inflammatory effect is not through reducing macrophage infiltration into white adipose tissue rather than switching macrophage phenotype from pro-inflammatory to anti-inflammatory phenotype. In addition to macrophages, it has been shown that CD8+ T cells accumulate in adipose tissue in obese mice and play an important role in adipose tissue inflammation^41,42^. Interestingly, CD47ASO treatment reduced CD8 expression in eWAT, suggesting that CD8+ T cells might be reduced in adipose tissue. Collectively, our data suggest that CD47ASO treatment may regulate both macrophage and T cell function, contributing to the reduced adipose tissue inflammation and improved glucose homeostasis.

In summary, our studies demonstrate that CD47ASO treatment stimulates physical activity, leading to increased energy expenditure and weight loss in obese mice. CD47ASO treatment also reduces obesity- associated metabolic complications including decreased adipose tissue inflammation and hepatosteatosis, and improved glucose tolerance. The results from this study suggest that CD47ASO may serve as a new therapeutic option for obesity and its related comorbidities.

## Methods

### Animals

All the experiments involving mice conformed to the National Institutes of Health Guide for the Care and Use of Laboratory Animals and were approved by the University of Kentucky Institutional Animal Care and Use Committee (IACUC). The authors compiled with the ARRIVE guidelines. All animals were housed in a pathogen-free environment at 22 °C in 14-h light/ 10-h dark cycle. Male six-week old C57BL6 mice (The Jackson Laboratory; Bar Harbor, ME) were fed with high fat diet (60% fat) (D12492, Research Diets, Inc, NJ) for 6 weeks to induce obesity and glucose intolerance. These diet-induced obesity (DIO) mice were then divided into three groups (n = 5–7 mice/group) and injected twice a week with saline, control ASO (GGCCAATACGCCGTCA) or CD47 ASO (ATTGATTAAGTCTGAG, targeting to mouse CD47) (provided by Ionis Pharmaceuticals, Carlsbad, CA) at a 25 mg/kg body weight by i.p. for 8 weeks with continuous high fat diet feeding in all groups. These ASOs are chemically stabilized with constrained ethyl modification in the wings and phosphorothioate linkages^43^. At the end of study, mice were anesthetized by intraperitoneal injection of Ketamine (100 mg/kg) combined with Xylazine (10 mg/kg). After blood was drawn and several tissues were harvested, cervical dislocation of mice was performed to ensure their deaths.

Another study was conducted with C57BL6J-Lep^ob^/Lep^ob^ (*ob/ob*) mice. Five-week old *ob/ob* mice were purchased from Jackson Laboratories. After one-week acclimation, *ob/ob* mice were divided into three groups and treated with saline, control ASO and CD47 ASO at 25 mg/kg body weight (i.p) twice a week for 8–9 weeks.

### Metabolic analysis

Body weight was measured weekly. At the end of the study, body composition was measured by Echo MRI^13^. Two weeks prior to the end of study, mice were placed in Sable Promethion system with in-cage running wheels individually for 5 days for measurement of food intake, water intake and indirect calorimetry.

### Cold exposure and body temperature measurement

Implantable Programmable Temperature Transponder 300 (IPTT-300, BioMedic Data Systems, Delaware, USA) was interscapularly implanted into mice. After one day of acclimatization, mice were put in 4 °C cold room individually without food for 6 h. Body temperature of the mice was monitored at 0, 1, 2, 3, 4 and 6 h during cold exposure.

### Blood parameter analysis and glucose tolerance test

At the end of study, blood samples were obtained via cardiac puncture. Plasma alanine aminotransferase (ALT) and aspartate aminotransferase (AST) levels were measured by using ALT and AST assay kit (Connecticut, USA). Blood glucose levels were measured by using glucometer. Plasma insulin levels were measured by using ELISA Kit (Crystal Chem USA, IL). For glucose tolerance test (GTT), mice were fasted 6 h before intraperitoneal injections of glucose (1 g/kg body weight). Blood glucose concentrations were measured using a glucometer at 0, 15, 30, 60, and 120 min post injection.

### Lipid analysis

Plasma total cholesterol and triglyceride, and hepatic cholesterol and triglyceride concentrations were determined enzymatically with Wako kits (Richmond, USA). For analysis of hepatic lipid, approximately 50 mg of liver was placed into 500 μl of chilled Krebs Ringer Phosphate buffer (118 mM NaCl, 5 mM KCl, 13.8 mM CaCl_2_, 1.2 mM MgSO_4_, 0.016% KH_2_PO_4_, 0.211% NaHCO_2_) and each sample was sonicated for ten times (3 s/time).

### White adipose tissue cAMP and cGMP measurement

cAMP and cGMP levels in white adipose tissue were measured by using the cAMP and cGMP ELISA kits from R&D Systems (Minneapolis, USA) and Abcam (Boston, USA), respectively. In brief, 100 mg of epididymal adipose tissue was homogenized in 1 ml of 0.1N HCl and the supernatant was collected. The samples were then neutralized with 1N of NaOH or neutralizing reagents provided from the kits. cAMP or cGMP levels in the supernatant were measured and calculated based on the cAMP or cGMP standard curve following the instruction manual.

### Western blotting

Liver tissues were homogenized in RIPA buffer (Sigma, St. Louis, MO, USA) plus protease and phosphatase inhibitors (Pierce, Waltham, MA, USA). After concentrations were measured using a BCA Assay (Pierce, Waltham, MA, USA), 30 μg protein/well was subjected to SDS-PAGE gel under reducing conditions and transferred onto a nitrocellulose membrane. After blocking, the membrane was incubated with anti-β-actin (1:5000 dilution; Santa Cruz, Dallas, TX, USA) and anti-CD47 (1:1000 dilution; Abcam, Cambridge, MA, USA) at 4 °C overnight. After washing, the membrane was incubated with horseradish peroxidase-conjugated secondary antibodies (Jackson Labs, Bar Harbor, ME, USA). The reaction was visualized by using an enhanced chemiluminescence system (Pierce, Waltham, MA, USA).

### Tissue histology

Fat tissues and liver tissues from all groups of animals were fixed in 10% Formalin for 24 h, embedded in paraffin, sectioned at 5 μm, and stained with hematoxylin and eosin-stain (H&E) by standard method by using the service from COBRE Pathology Core at University of Kentucky. In addition, liver cryo-sections were fixed with 4% PFA and stained with 0.5% Oil red O in 60% isopropyl alcohol. All images were acquired with a Nikon Eclipse 55i.

### Real-time quantitative PCR

Total RNA from tissues were extracted using TRIzol Reagent (Thermo Fisher Scientific, Massachusetts, USA). RNA was reverse transcribed to cDNA by the cDNA Synthesis Kit (Quantabio, Massachusetts, USA). Real-time quantitative PCR was performed using a MyiQ Real-time PCR Thermal Cycler (Bio-Rad) with SYBR Green PCR Master Kit (Qiagen, Valencia, CA). Relative mRNA expression was calculated using the MyiQ system software as previous reported^44^ and normalized to β-actin levels. All primer sequences utilized in this study are found in Table 1.

**Table 1:** Mouse primer Sequences for QPCR.

Gene	Primer sequence	Genes	Primer sequence
FASN	5’-TCCTGGAACGAGAACACGATCT-3’ 5’-GAGACGTGTCACTCCTGGACTTG-3’	SCD-1	5’-TTCTTGCGATACACTCTGGTGC-3’ 5’-CGGGATTGAATGTTCTTGTCGT-3’
ACC	5’-CCCAGCAGAATAAAGCTACTTTGG-3’ 5’-TCCTTTTGTGCAACTAGGAACGT-3’	SREBP1c	5’-GGAGCCATGGATTGCACATT-3’ 5’-ACAAGGGTGCAGGTGTCACC-3’
HMGCR	5’-TGCACGGATCGTGAAGACA-3’ 5’-GTCTCTCCATCAGTTTCTGAACCA-3’	HMGCR	5’-TGCACGGATCGTGAAGACA-3’ 5’-GTCTCTCCATCAGTTTCTGAACCA-3’
F4/80	5’-CTTTGGCTATGGGCTTCCAGTC-3’ 5’-GCAAGGAGGACAGAGTTTATCGTG-3’	IL-1β	5’-TGGAGAGTGTGGATCCCAAGCAAT-3’ 5’-TGTCCTGACCACTGTTGTTTCCCA-3’
TNFα	5’-AGCCGATGGGTTGTACCT-3’ 5’-TGAGTTGGTCCCCCTTCT-3’	CD4	5’-TGGCTCAGCTCAACAATAC-3’ 5’-GGCAGCAGCGAAGAATAA-3’
CD8	5’-AAGAAAATGGACGCCGAACTT-3’ 5’-AAGCCATATAGACAACGAAGGTG-3’	NOS2	5’-AAGAGGAGCAACTACTG-3’ 5’-GCTCTGTTGAGGTCTAA-3’
Arg1	5’-CTCCAAGCCAAAGTCCTTAGAG-3’ 5’-AGGAGCTGTCATTAGGGACATC-3’	UCP1	5’-ACTGCCACACCTCCAGTCATT-3’ 5’-CTTTGCCTCACTCAGGATTGG-3’
UCP2	5’-GGCTGGTGGTGGTCGGAGAT-3’ 5’-CCGAAGGCAGAAGTGAAGTG-3’	UCP3	5’-ACCTGGACTGCATGGTAAGG-3’ 5’-GAGAGCAGGAGGAAGTGTGG-3’
CPT1α	5’-CTCTATGTGGTGTCCAAG-3’ 5’-CACAGGACACATAGTCAG-3’	CPT1β	5’-ACCTGAGCTGTGCTGAATAAA-3’ 5’-ACAGGAGACGGACACAGATA-3’
CD36	5’-TTGTACCTGGGAGTTGGCGAGAAA-3’ 5’-ACAGTTCCGATCACAGCCCATTCT-3’	PGC-1α	5’-CTGCATGAGTGTGTGCTGTG-3’ 5’-CAAATATGTTCGCAGGCTCA-3’
CD31	5’-TGCAGGCATCGGCAAAG-3’ 5’-GCATTTCGCACACCTGGAT-3’	COX1	5’-CGTAACTGCCCATGCTTT-3’ 5’-CTGCTCCTGCTTCTACTATTG-3’
ATGL	5’-AACACCAGCATCCAGTTCAA-3’ 5’-GGTTCAGTAGGCCATTCCTC-3’	HSL	5’-GGCTCACAGTTACCATCTCACC-3’ 5’- GAGTACCTTGCTGTCCTGTCC-3’
β-Actin	5’-GGCTGTATTCCCCTCCATCG-3’ 5’-CCAGTTGGTAACAATGCCATGT-3’	mtDNA	5’-CCGCAAGGGAAAGATGAAAGAC-3’ 5’-TCGTTTGGTTTCGGGGTTTC-3’
nDAN	5’-GCCAGCCTCTCCTGATTTTAGTGT-3’ 5’-GGGAACACAAAAGACCTCTTCTGG-3’		

### Statistical analysis

Statistical analysis was performed using Prism 9 (GraphPad Software, San Diego, CA). All data are presented as the mean ± SEM. Statistical significance between two groups was determined using two-tailed Student’s *t*-test. One-way ANOVA followed by Bonferroni’s multiple comparisons test or 2-way ANOVA followed by Tukey’s multiple comparisons test was applied for multi-group comparisons. *P* values of less than 0.05 were considered to be significant.

## Supplementary Information

Supplementary Information.

## Data Availability

The data that support the findings of this study are available from the corresponding author upon reasonable request.
