# Supplementary material for: CD47 antisense oligonucleotide treatment attenuates obesity and its-associated metabolic dysfunction
Source: Sci Rep. 2023 Feb 16;13:2748. doi: 10.1038/s41598-023-30006-2 (PMC9935863; doi:10.1038/s41598-023-30006-2)

CD47 antisense oligonucleotide treatment attenuates obesity and its-associated metabolic dysfunction

Taesik Gwag [1, 3#], Dong Li [1, 3#], Eric Ma [1], Zhenheng Guo [1], Ying Liang [2], and Shuxia Wang[1, 3 *]

[1]Department of Pharmacology and Nutritional Sciences, [2]Department of Toxicology and Cancer Biology, University of Kentucky, Lexington, KY 40536, [3] Lexington Veterans Affairs Medical Center, Lexington, KY 40502

Fig. S1: Six weeks of high fat diet feeding induced obesity and glucose intolerance in male C57BL6 mice

Male 6-week old C57BL6 mice were fed with LF or HF diet for six weeks. (A). Body weight; (B). Glucose tolerance test (GTT) and area under the curve were analyzed. Data are represented as mean ± SE (n=5-7 mice/group). *** $P<0.001$

Fig. S2: Two additional CD47ASOs treatment induced weight loss and reduced adiposity in diet-induced obese mice

Two additional CD47ASOs (targeting to mouse CD47: CD47ASO1 with sequence of ACGATTTGTTCAACTT and CD47ASO2 with sequence of TACGATTTGTTCAACT) were administrated into male diet induced obese mice (DIO) at dosage of 25 mg/kg (i.p. twice a week) for 8 weeks with continuous high fat feeding. (A). CD47 mRNA levels from different tissues after saline or ASOs treatment by qPCR; (B). Weekly body weight and body weight change after ASO treatment; and (C) Body composition (fat mass and lean mass) detected by EchoMRI. Data are represented as mean ± SE (n=4-7 mice/group). *$P<0.05$, ** $P<0.01$, *** $P<0.001$

Fig. S3: CD47ASO treatment induced weight loss, improved glucose tolerance and fatty liver disease in *ob/ob* mice

CD47ASO or control ASO was administrated into male five-week old *ob/ob* mice at dosage of 25mg/kg (i.p twice a week) for 9 weeks. (A). Weekly body weight after ASO treatment; (B). Body composition (fat mass and lean mass) detected by EchoMRI; (C). Glucose tolerance test (GTT) and area under the curve were analyzed; and (D). Representative liver H&E staining and live triglyceride levels. Data are represented as mean ± SE (n=4-7 mice/group). **P*<0.05, ** *P*<0.01, *** *P*<0.001, *****P*<0.0001

Fig. S4: Effect of CD47ASO treatment on white fat browning or brown fat activity

(A). Representative H&E staining of inguinal white fat (iWAT) and gene expression by qPCR; (B). Representative H&E staining of brown fat (BAT) and gene expression by qPCR; and (C). Body temperature measurement during cold exposure (at 4^0 C).

Full-length blots for Figure 1C: CD47ASO treatment induced weight loss and reduced adiposity in diet-induced obese mice

Suppl Fig. S1

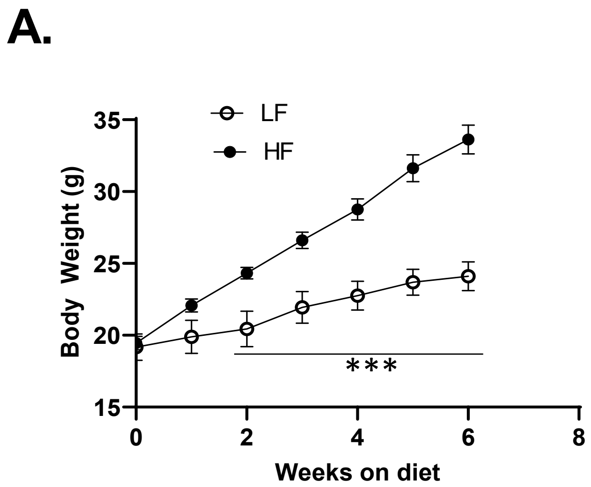

A.
LF
HF
Body Weight (g)
35
30
25
20
15
0
2
4
6
8
Weeks on diet
***

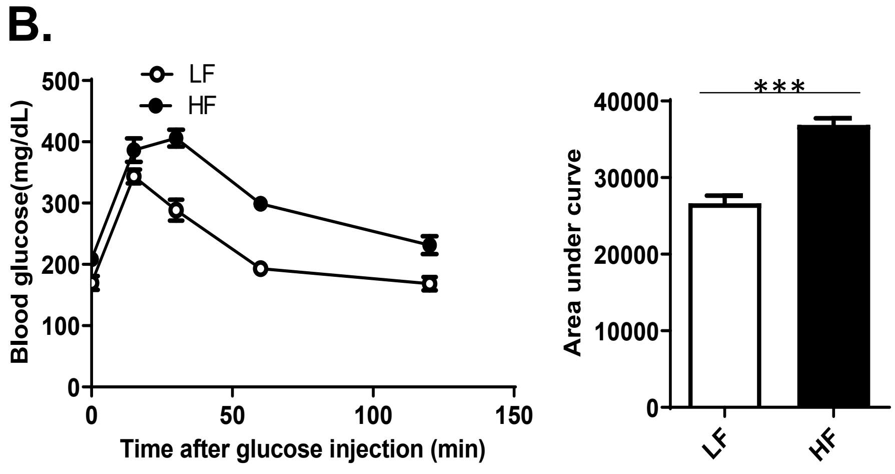

B.
LF
HF
Blood glucose(mg/dL)
500
400
300
200
100
0
50
100
150
Time after glucose injection (min)
Area under curve
40000
30000
20000
10000
***
LF
HF

Suppl Fig. S2

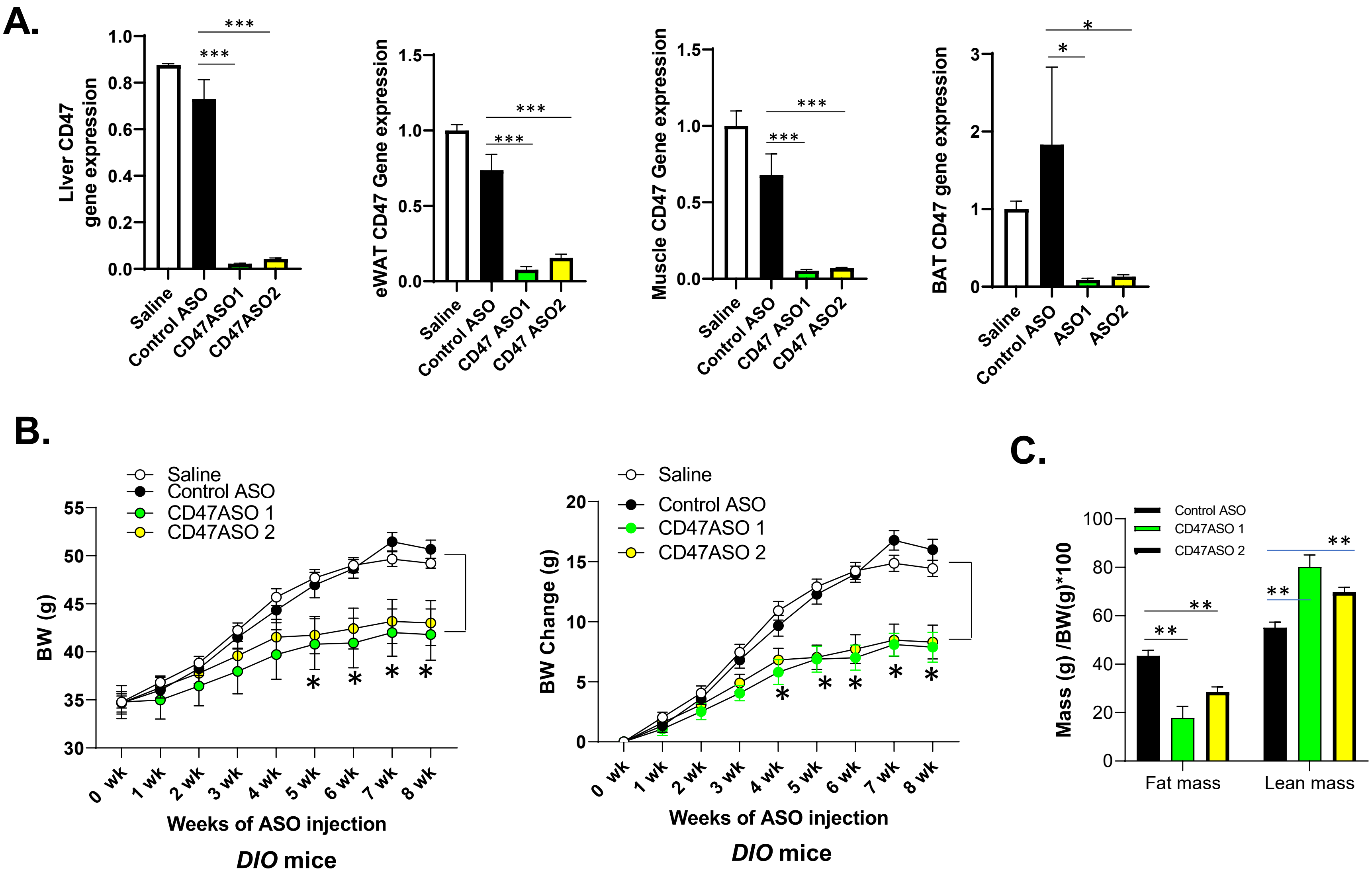

A.
LIver CD47 gene expression
1.0
0.8
0.6
0.4
0.2
0.0
***
***
Saline
Control ASO
CD47ASO1
CD47ASO2
eWAT CD47 Gene expression
1.5
1.0
0.5
0.0
***
***
Saline
Control ASO
CD47 ASO1
CD47 ASO2
Muscle CD47 Gene expression
1.5
1.0
0.5
0.0
***
***
Saline
Control ASO
CD47 ASO1
CD47 ASO2
BAT CD47 gene expression
3
2
1
0
*
*
Saline
Control ASO
ASO1
ASO2
B.
Saline
Control ASO
CD47ASO 1
CD47ASO 2
BW (g)
55
50
45
40
35
30
* * * *
0 wk
1 wk
2 wk
3 wk
4 wk
5 wk
6 wk
7 wk
8 wk
Weeks of ASO injection
DIO mice
Saline
Control ASO
CD47ASO 1
CD47ASO 2
BW Change (g)
20
15
10
5
0
* * * * *
0 wk
1 wk
2 wk
3 wk
4 wk
5 wk
6 wk
7 wk
8 wk
Weeks of ASO injection
DIO mice
C.
Control ASO
CD47ASO 1
CD47ASO 2
Mass (g) /BW(g)*100
100
80
60
40
20
0
**
**
**
**
Fat mass
Lean mass

Suppl Fig. S3

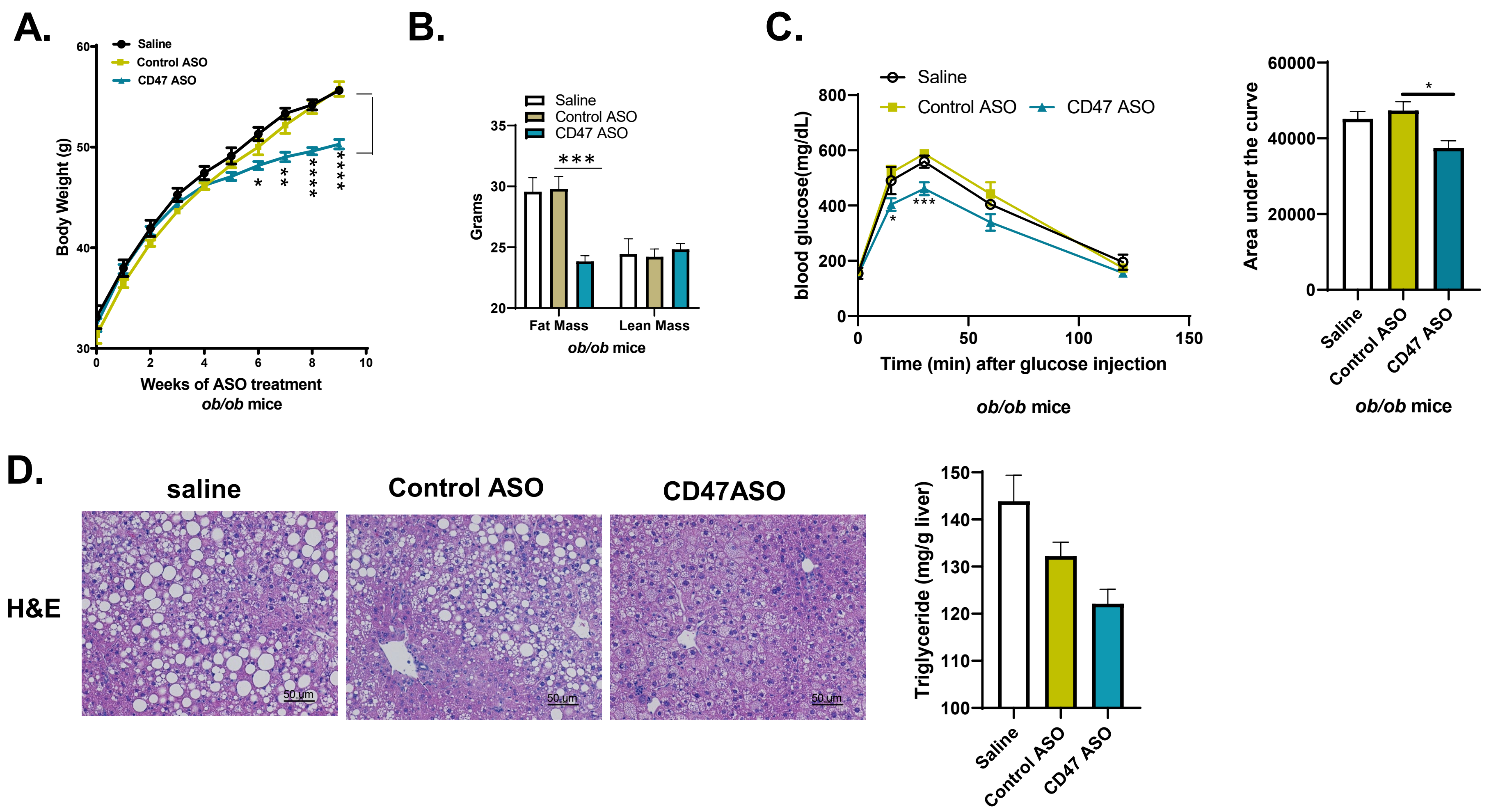
A.
Saline
Control ASO
CD47 ASO
Body Weight (g)
Weeks of ASO treatment
ob/ob mice
B.
Saline
Control ASO
CD47 ASO
Grams
***
Fat Mass
Lean Mass
ob/ob mice
C.
Saline
Control ASO
CD47 ASO
blood glucose(mg/dL)
Time (min) after glucose injection
ob/ob mice
Area under the curve
Saline
Control ASO
CD47 ASO
ob/ob mice
D.
saline
Control ASO
CD47ASO
H&E
50 µm
Triglyceride (mg/g liver)
Saline
Control ASO
CD47 ASO

Suppl Fig. S4

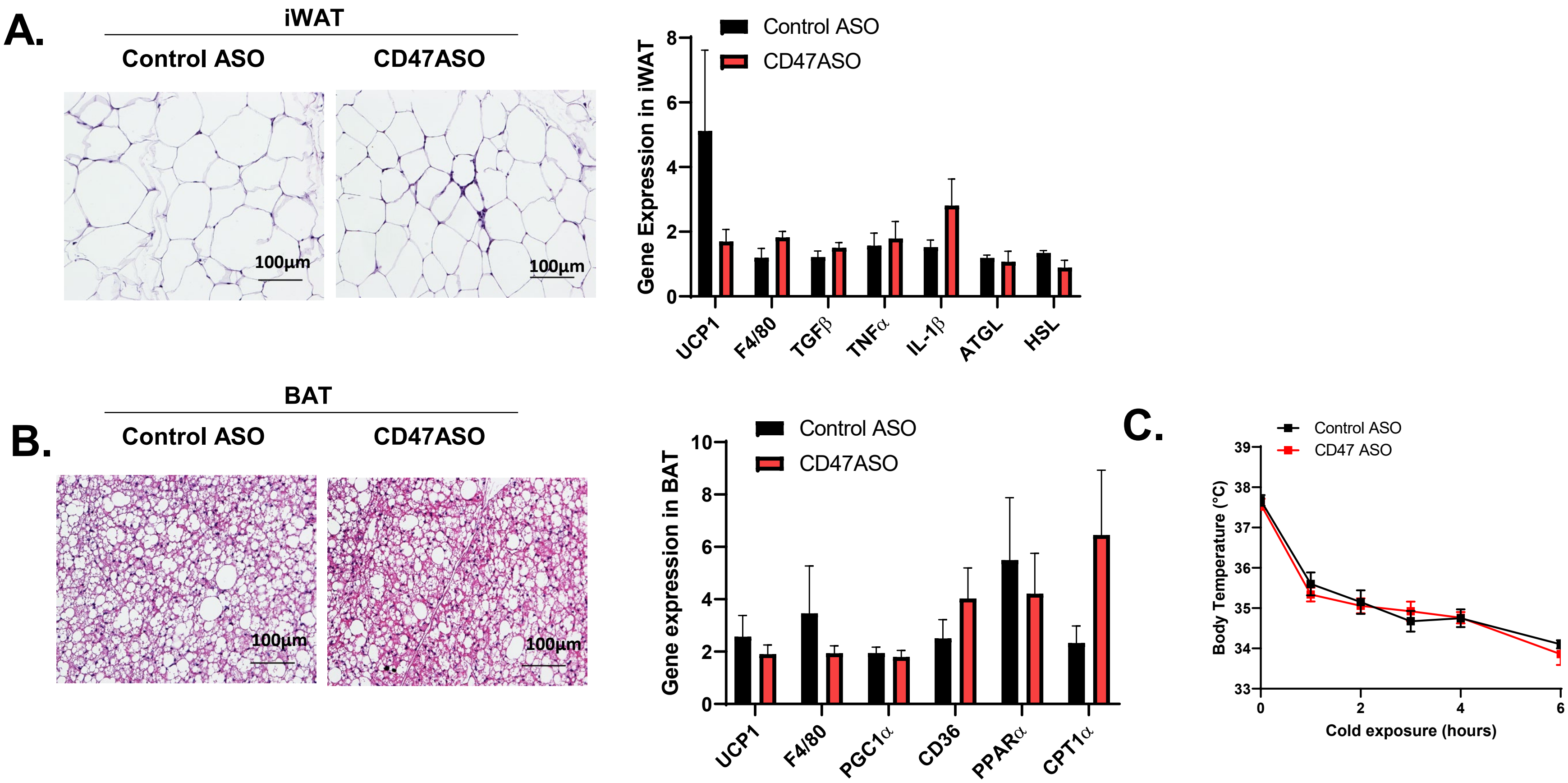

A.
iWAT
Control ASO
CD47ASO
100µm
100µm
Control ASO
CD47ASO
Gene Expression in iWAT
UCP1
F4/80
TGFβ
TNFα
IL-1β
ATGL
HSL
B.
BAT
Control ASO
CD47ASO
100µm
100µm
Control ASO
CD47ASO
Gene expression in BAT
UCP1
F4/80
PGC1α
CD36
PPARα
CPT1α
C.
Control ASO
CD47 ASO
Body Temperature (°C)
Cold exposure (hours)

Full-length western blots for Figure 1C

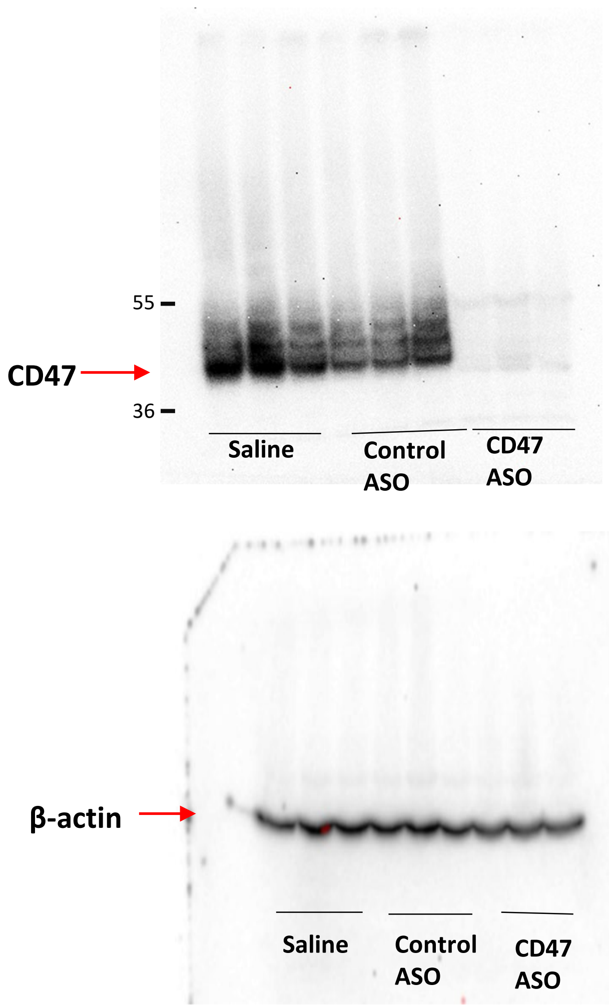

Full-length western blots for Figure 1C

(Different exposure time)

30 sec

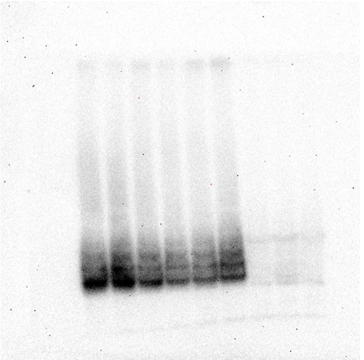

5 min

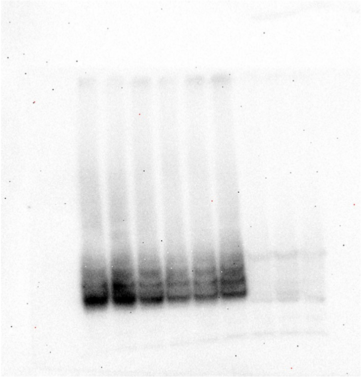

10 min

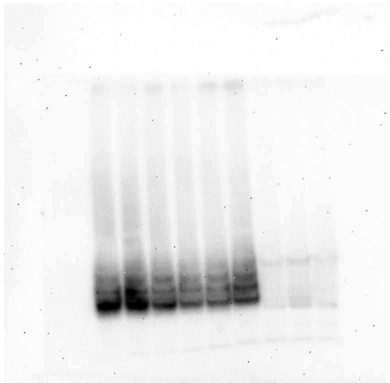

Supplement: Supplementary file 1 — Supplementary Information. [file 41598_2023_30006_MOESM1_ESM.pdf]
